# From unimodal to multimodal dynamics of verbal and nonverbal cues during unstructured conversation

**DOI:** 10.1371/journal.pone.0309831

**Published:** 2024-09-25

**Authors:** Tifenn Fauviaux, Ludovic Marin, Mathilde Parisi, Richard Schmidt, Ghilès Mostafaoui

**Affiliations:** 1 EuroMov Digital Health in Motion, Univ Montpellier, IMT Mines Ales, Montpellier, France; 2 College of the Holy Cross, Worcester, MA, United States of America; 3 CY Cergy Paris Université - ETIS UMR 8051, Cergy, Pontoise, France; University of Missouri Columbia, UNITED STATES OF AMERICA

## Abstract

Conversations encompass continuous exchanges of verbal and nonverbal information. Previous research has demonstrated that gestures dynamically entrain each other and that speakers tend to align their vocal properties. While gesture and speech are known to synchronize at the intrapersonal level, few studies have investigated the multimodal dynamics of gesture/speech between individuals. The present study aims to extend our comprehension of unimodal dynamics of speech and gesture to multimodal speech/gesture dynamics. We used an online dataset of 14 dyads engaged in unstructured conversation. Speech and gesture synchronization was measured with cross-wavelets at different timescales. Results supported previous research on intrapersonal speech/gesture coordination, finding synchronization at all timescales of the conversation. Extending the literature, we also found interpersonal synchronization between speech and gesture. Given that the unimodal and multimodal synchronization occurred at similar timescales, we suggest that synchronization likely depends on the vocal channel, particularly on the turn-taking dynamics of the conversation.

## Introduction

Social interaction, such as face-to-face communication, can be mirrored as a complex choreography wherein each speaker mutually exchanges perceptual information through the vocal (auditory) and visual channels [1–4].

Through the vocal channel, speech encompasses a combination of verbal and nonverbal expressions. Verbal cues convey the linguistic and semantic meaning of words whereas nonverbal (i.e., suprasegmental) speech features are used to communicate structural information and emotion [5, 6]. Nonverbal speech features refer to prosody. Prosody involves fluctuations in fundamental frequency, amplitude, duration of segments and syllables, and intervals of pauses [7]. The visual channel comprises all elements of nonverbal behaviors, including body gestures [8]. Often, they accompany the speech’s content (i.e., co-speech gestures) to perform some specific communicative function such as emotional expression, agreement, or disagreement [9]. Co-speech gestures consist most often of manual and head movement and represent the gestures the most studied in the literature [10, 11].

Both prosody and co-speech channels are beneficial for the conversation conveying respectively 38% and 55% of the information between the speaker and listener [5]. Their respective functions complement or supplement each other. Prosody is leveraged by the speaker as a means to express his/her beliefs, attitudes, and emotions concerning the content of the message [12]. The speaker also uses co-speech head and hand gestures as a visual aid to clarify complex concepts that may be challenging to convey solely through vocal communication (i.e., pointing at an object while saying, “What is that?”) [13]. These co-speech gestures also signal the speaker’s desire to keep talking. For the listener, nonverbal gestures (i.e., head nods) and brief vocalizations (i.e., “mm hm,” “uh-huh,” “yeah”) can be used as backchannels to convey information about his/her continued involvement in the conversation or to signal his/her desire to speak next [14]. Most of the time, the speaker and the listener combine modalities (i.e., a pointing gesture while speaking; a head nod combined with a “yeah”) [15].

Multimodality plays an essential role in orchestrating the turn-taking of the conversation [16–18]. Turn-taking is a fundamental structural feature of conversation [19]. In most cases, only one individual speaks at a time, taking on the role of the speaker, while an interacting partner listens, taking on the role of the listener [20]. Conversation is then associated with rapid, natural alternations of turn-taking with minimal overlap and speech interruptions [21–23]. This spontaneous way in which individuals naturally take their turn to speak stands out as one of the conversation’s outstanding features. The management of turn-taking relies on a variety of complex signals, including prosodic cues and nonverbal cues [24]. Therefore, to guarantee the fluidity of the turn-taking, a coordinated dance of vocal and nonverbal signals must be established [1, 25, 26].

Within this coordinated dance, each individual engages in a “back and forth” exchange of communicative signals, operating with their distinctive frequency and rhythm [27]. During interactions, the communicative signals will coordinate with each other. Coordination, in this context of verbal and nonverbal information exchange, refers to the concept of individuals mutually influencing each other’s behavior over time [28]. Over the past few decades, research has explored how coordination functions in communication and language interaction.

Early theories, such as the Communication Accommodation Theory postulated that speakers accommodate their communicative behavior toward and away from each other to indicate their attitude, either through convergence (i.e., individuals accommodate their linguistic, paralinguistic, and nonverbal features to become more similar) or divergence (i.e., individuals differentiate their speech patterns and nonverbal cues compared to those of others) [29].

Another perspective is the interactive alignment theory by Pickering and Garrod [30] which explains how individuals share mental representation by automatically aligning their linguistic (syntactic and linguistic) behaviors and situational models [31]. Relying on a priming mechanism, alignment at one level, such as lexical, influences and extends to other levels, such as prosodic and syntactic [32].

Fusaroli et al. [32] argued that alignment is only one of several mechanisms used to manage linguistic processing. Building on this, they proposed an alternative approach to better characterize the complexity of multimodal conversation, viewing dialogue as an interpersonal synergy. Dialog interactions are proposed to be dynamic and context-sensitive, where interlocutors assume complementary roles completing linguistic behaviors rather than copying them. Therefore, dialogs cannot be understood at the level of the individual alone but rather within the functional system level of the dyad.

A proposed extension to these theories relates to the concept of complexity matching found in dyadic conversation [33, 34]. This alternative framework views individuals in conversation as two complex networks, where each of their behaviors, such as speech and movement, exhibit multiscale dynamics [26, 35]. It investigates the matching of behaviors in terms of statistical, global dynamics rather than focusing on specific actions [33]. When two individuals match their level of complexity, they maximize their exchange of information [35]. These matching behaviors follow power law distributions, indicative of the multiscale variations characteristic of complex systems [36].

**Fig 1:**
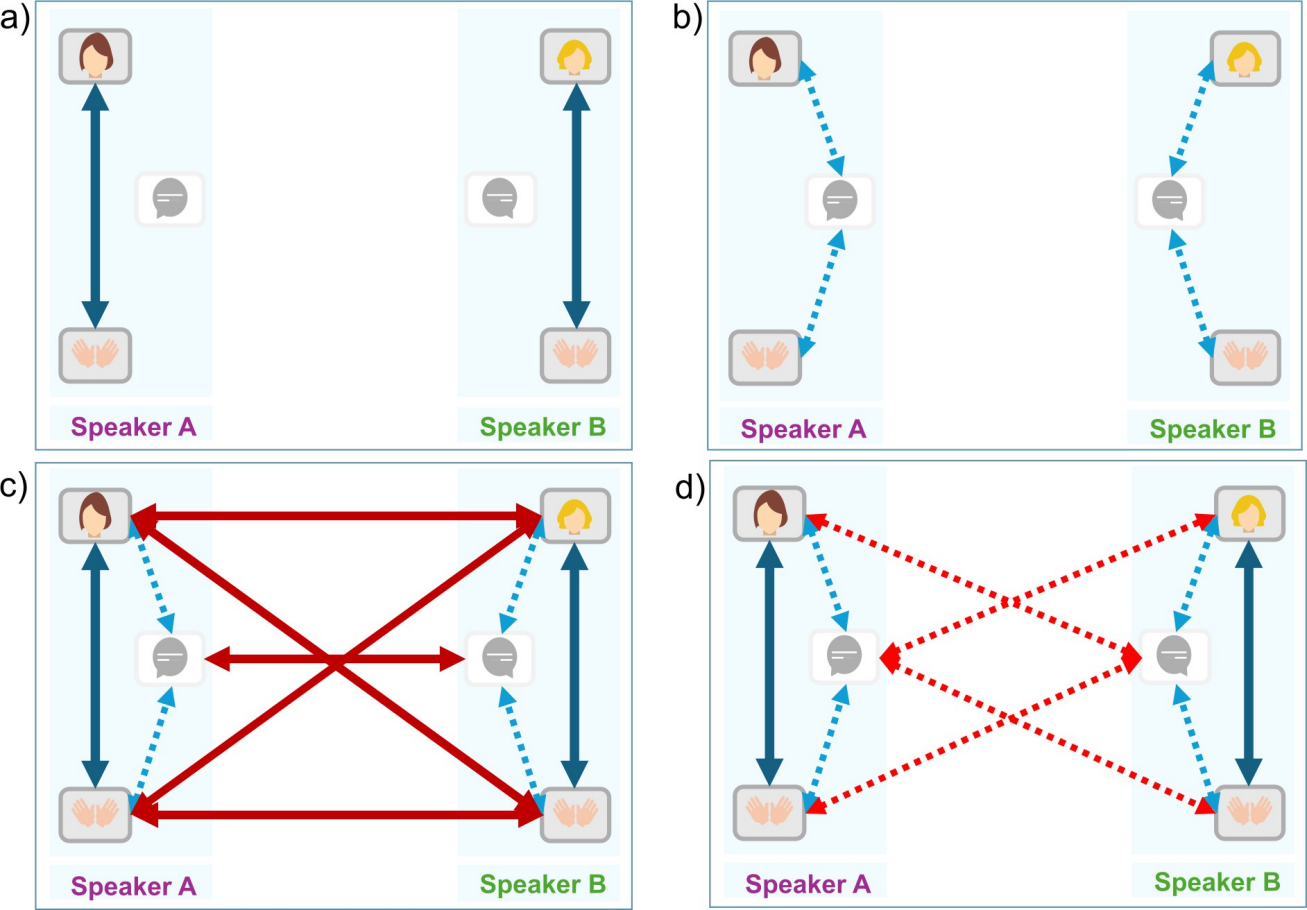
Schematic diagram of the relationship between intrapersonal and interpersonal synchronization, whether unimodal or multimodal. A) and B) represent intrapersonal synchronization among the modalities of a single speaker. A) Full blue arrows highlight the unimodal relationship between the gestures generated by a single individual (i.e., head vs. head, head vs. wrist). B) Dashed blue arrows highlight the multimodal relationship between the voice and the gesture produced by a single individual (i.e., head vs. voice; wrist vs. voice). C) and D) represent the interpersonal synchronization between the modalities of speaker A and speaker B. C) Full red arrows highlight the unimodal relationships between the movements of speaker A and speaker B, and between their voices (i.e., head vs. head, head vs. wrist, voice vs. voice). D) Dashed red arrows highlight the multimodal relationships between the voice of speaker A and the gesture of speaker B and inversely (i.e., head vs. voice; wrist vs. voice).

The idea of defining the behaviors of individuals within the interaction as a global dyadic system that represents an interpersonal synergy structure is part of the behavioral dynamics perspective in which coordinated signals can be seen as an entrainment process of biological and behavioral rhythms [37, 38]. Researchers from this perspective have shown that coordination in social interaction is governed by the same dynamical processes of self-organization that constrain interaction interacting physical oscillators [39, 40]. These principles represent a universal self-organizing law that occurs at multiple scales (from neural to behavioral, or social scales of nature) [41]. Synchronization emerges as the outcome of both space and time coordination resulting in a rhythmic convergence of behavior, whether in-phase (behaviors flowing in the same direction) or anti-phase (behaviors flowing in opposite directions) [42]. For example, during the conversation, while in-phase coordination could be illustrated by the two interlocutors nodding their heads simultaneously in agreement, the anti-phase relationship would imply each speaker nodding their head in an alternating fashion. These patterns of synchronization, alongside the degree of similarity in individuals’ behavior, serve as fundamental measurements of synchronization [42]. Studies have shown that synchronization can exhibit itself through two interconnected phenomena: self-synchrony within individuals (i.e., intrapersonal synchronization) and interpersonal synchronization between individuals (Fig 1) [43]. Moreover, synchronization between signals of different modalities has received significant interest in past years [44]. In this study, we define modality as referring to the distinct nature of signals, such as movement-based signals and voice-based signals. Unimodal studies focused on a single modality, examining either speech synchronization or nonverbal gesture synchronization separately. In contrast, multimodal studies investigated the synchronization between two different modalities, specifically the synchronization between speech and gestures (Fig 1). These studies on synchronization are crucial, as they shed light on the fundamental basis of synchronization in facilitating social bonding [45]. In the following, we review studies on the unimodal analysis of speech, unimodal analysis of movements, and multimodal analysis of gesture and speech.

Unimodal analyses of speech focus on the interpersonal relationship between the speech of two interlocutors. Studies emphasized that when two individuals are engaged in a conversation, they tend to become similar in how they speak. Many studies have documented this phenomenon of similarity through different terms such as prosodic accommodation, convergence, or acoustic-prosodic entrainment, e.g. [46]. However, one must be careful with the generic term entrainment, as it differs from the ones used in the behavioral dynamics field. Here, entrainment refers to the similarity or alignment of prosodic speech features (pitch, loudness, intensity, speech rate…) between speakers at the conversation or the turn level [47]. In this field of speech communication, synchrony is considered a phenomenon underlying similarity [46]. From a behavioral dynamics perspective, synchrony refers to the amount of similarity in prosodic behavior between speakers, such as when a speaker changes his/her voice intensity, his/her interlocutor reacts in a parallel way across time [47]. To confirm the dynamic display of similarity, synchrony can be assessed utilizing Pearson correlation, either at the level of individual turns or within a defined moving time window [46]. At the turn level, past studies analyzed the synchronization of spontaneous dyadic conversation looking at the dimensions of intensity, pitch, voice quality, and speaking rate across time. They found that all these features exhibit significant synchrony [47, 48]. De Looze et al. [46] investigated the evolution of prosodic synchrony with moving window time and found that prosodic synchrony varies several times throughout the conversation. They found that the fundamental frequency (F0) showed a greater amount of similarity compared to intensity, which displayed a lower occurrence of similarity. They suggested that high synchrony could be indicative of overlapped speech while lower synchrony might reflect the conversational dynamic where one individual is speaking while the other remains mostly silent.

The same process is found in the unimodal synchronization of non-verbal signals, both at the intrapersonal and interpersonal levels. Indeed, in the framework of motor synchronization, a set of studies has shown that individuals tend to coordinate their behavior. At the intrapersonal level, self-synchrony was first studied by Kelso [49] and Turvey et al. [50] through bimanual coordination and focus was then extended to coordination during walking [51], or between the movements of the hand and foot [52]. At the interpersonal level, Schmidt et al. [53] characterized the emergence of intentional coordination when they asked two participants to visually coordinate their legs in-phase or in antiphase with the rhythm of a metronome. This phenomenon was also found to happen unintentionally with individuals spontaneously coordinating their behaviors as soon as a perceptual coupling was established between them [54–56]. According to Bernieri and Rosenthal [57], interpersonal coordination can be described as either mimicry (or behavioral matching) or interactional synchrony. In this context, mimicry refers to the imitation of behaviors such as facial expressions, mannerisms, posture, and gestures, which do not require temporal coordination. For example, during a conversation, people might unintentionally mimic each other’s body language, like crossing their legs or touching their hair within a short window of time (and not necessarily at the same time). Synchronization, however, involves coordination in both space and time [58]. An illustration of this would be two people walking side-by-side, spontaneously synchronizing their step pattern at the same time. More recently, studies have focused on nonverbal behaviors during more naturalistic settings. In a series of knock-knock jokes, Schmidt et al. [41] uncovered a higher-than-expected level of synchronization between the bodily movements of the joke teller and the joke responder. The same synchronization results were likewise identified during unstructured conversations [59]. Moreover, Hale et al. [60] highlighted that coordination could also be accounting for more specific body parts, such as between the head movements of two interlocutors.

In addition to the investigation of unimodal synchronization, research has also explored the field of multimodal coordination between voice and gesture at the intrapersonal level. Condon [61] was the first to delve into this area, discussing self-synchrony as the representation of a coordinated system between the speaker’s speech and gesture [43]. From these observations, several researchers have noted that speech and gesture are temporally synchronous: the rhythmic pulse of prosody events such as stressed syllables and temporal patterns of nonverbal gestures are influencing each other [10, 62]. For example, Pouw et al. [63] reported that a sudden increase in speech intensity will entrain spontaneous co-speech hand gestures. However, while intrapersonal synchronization appears to be finely tuned at the prosodic level with gesture-speech coupling occurring on relatively short timescales [64], less is known about the interpersonal coupling between the gesture and the voice.

To our knowledge, only a few studies worked on the synchronization of speech and gesture at the dyad level. Paxton and Dale [65] investigated the multimodal coordination between speech and bodily movements during an affiliative and an argumentative interaction. They found that coordination indeed occurs between the speaking event of one participant and the movement of the listener but dropped for argumentative conversation. However, nonverbal gestures were assessed globally, considering the overall body motion whereas speech was only considered as binary on/off events. Paxton et al. [36] created networks of speech and movement to highlight the interconnectivity of these modalities during a cooperative task where participants had to build the tallest tower structure possible. The authors analyzed patterns of influence between multimodal behaviors through the analyses of behavior matching (i.e., synchronization) and complexity matching. They found high cross-correlation values emphasizing synchrony in both speech and movement modalities, and observed a lower network strength, indicating efficient communication (i.e., a lower degree of connectivity between modalities). While investigating multimodal coordination during an interpersonal interaction, the type of task used in this study is not representative of daily social interaction and the structure imposed by such a task could have influenced the pattern of synchronization. More recently, Trujillo et al. [66] specifically focused on analyzing the correlation between linguistic alignment and movement alignment during a task-oriented conversation and an affiliative conversation. They found that movement entrainment was positively correlated with lexical entrainment but negatively with semantic entrainment. However, the authors concentrated their analyses on correlations between voice-to-voice and movement-to-movement interactions, rather than exploring all possible combinations of modalities.

While most of the research on synchronization focuses either on motor behavior or speech dynamics independently, studies exploring the interpersonal dynamics of speech in relation to nonverbal gestures remain limited [41, 59, 67]. A more thorough account of these interpersonal multimodal dynamics is needed as their synchronization is a foundation for effective social rapport [45]. Moreover, as pointed out by Alviar et al. [34], the precise timescale of the different patterns of coordination between modalities should be studied both at the level of the individual as well as at the level of the dyad interaction. Consequently, the current study aims to fill this gap by analyzing the multimodal synchronization between prosodic features of speech and nonverbal head/hand gestures, both intra and interpersonal, during a dyadic conversation. For this purpose, we examined synchronization among an online dataset, where dyads engaged in an unstructured conversation (i.e., where turn-taking between the partners was not controlled) [68]. This dataset recorded the global motion of the dyads as well as their specific hand, head movement, and speech. Synchronization of speech and gesture was assessed using the methodology of the cross-wavelet transform [41, 67]. Especially, the cross-wavelet transform enabled us to extract two fundamental measurements of synchronization, namely, the pattern of synchronization and the degree of coherence between the two individuals’ dynamics, as emphasized previously. While the pattern of synchronization refers to how the signals, such as speech and gestures, align in time, the degree of coherence highlights how closely correlated or similar these signals are in terms of their frequency components [42]. These metrics were extracted at the intrapersonal and interpersonal levels, encompassing both unimodal and multimodal synchronization analyses of speech and gesture. Consequently, we decided to separate our work into two distinct questions:

1) What are the observed degree and patterns of synchronization at the intrapersonal level, encompassing both unimodal and multimodal synchronization?
  Based on previous studies, we hypothesize finding higher-than-chance coherence within both unimodal and multimodal modalities [67].
  While we expect to find the wrist leading the voice [69], we lack empirical evidence to formulate hypotheses for unimodal synchronization patterns.
2) Is this intrapersonal synchrony also related to the interpersonal synchrony between individuals’ movements and voices, both in terms of unimodal and multimodal synchronization?

We expect to find higher-than-chance synchronization between the participants’ movements and between the participants’ voices respectively [41, 46, 59]. However, we lack the theoretical foundations to formulate hypotheses about multimodal coordination. Similarly, we lack empirical evidence to formulate hypotheses for unimodal and multimodal synchronization patterns.

## Materials and methods

### Task and recording

The task and recording files come from the original dataset of Met Research available at https://github.com/facebookresearch/TalkingWithHands32M/ [68, 70]. The dataset consisted of 50 sessions of two people engaged in unstructured face-to-face conversations. Each of these sessions contained 1 to 4 discussions. These conversations lasted from 7 to 15 minutes. During the conversation, participants were free to talk about specific topics. The topic originated from talking points initially intended for informal conversations in English such as “Where are you planning to go for your next vacation?” or “What good restaurants do you know of around here?”. The specific role of the speaker and listener was not specified, and participants were free to engage in and drift to another topic [68]. The duration of each discussion varied, resulting in an average conversation time of about 11 minutes.

Motions were recorded in a room fitted with an Optitrack motion capture system. and built into Biovision Hierarchy files. The motion capture system’s cameras were positioned on each side of the two participants, as well as from below and above, to capture the overall body of each participant, resulting in 83 different body coordinates being acquired. The motion recording of each participant was then built into Biovision Hierarchy files. The audio was recorded using OctaMic XTC, a versatile preamplifier, and was synchronized with motion data by BrightEye 56 Pulse Generator [68].

Out of the thirty-two sessions that were at our disposal, only fourteen were selected for our analyses (i.e., as multiple discussions occurred within each session, it resulted in a total of thirty-two discussions). These fourteen specific sessions were chosen due to their inclusion of both audio and motion data from the presented tasks.

### Data processing

#### Motion analyses

We used a GitHub script available at https://github.com/wspr/bvh-matlab and a MATLAB script to extract specific body coordinates (X, Y, Z). All body data was processed at 90 fps. We applied a second-order Butterworth low pass filter to the data. Then, we calculated the velocity time series from this filtered position data. Of the 83-body part velocity data, the final analyses focused on a head marker, and the sum of the left and wrist marker velocities, as both can be used to accompany speech.

### Audio analyses

Every session from the dataset consisted of two distinct audio channel files (in WAV), intended to capture the voices of the participants who wore the microphones. Going through the analysis of each audio file, it became clear that both voices were indiscernible: Attributing a single audio signal exclusively to one participant was challenging since the responder’s voice was also captured in the recording. To overcome this issue, we used the ELAN software to manually annotate the participants’ speaking time [71].

To quantitatively assess the shared temporal structure of speech and gesture, we calculated the amplitude envelope of the speech. The amplitude envelope is a continuous measure for tracking the rhythmicity of speech (i.e. the prosody of speech) known to correlate highly with articulatory movements [72]. The amplitude envelope was extracted through the code of Pouw and Trujillo [73]. which computed the analytic signal and temporal fine structure using the Hilbert transform method.

We also used the speaking behavior annotations that we manually annotated along with a MATLAB script to describe the turn-taking behavior of the conversation. A turn is defined as a sequence of speech units from a single speaker, that can be separated by pauses, but which are not interrupted by speech units of the other speaker [74]. These speech units are characterized as speech segments from one speaker without any silence exceeding 200ms [24]. Therefore, to create these speech units, we merged speech segments that were separated by silence shorter than 200 milliseconds. The remaining silences, (longer than 200 milliseconds) can be identified as pauses, if they occurred between consecutive speech units from the same speaker, or as gaps if they occurred between consecutive speech units from different speakers. If pauses occurred without any detected voice from the other speaker, indicating no interruption, the speech units from the same speaker were combined to form a turn. For example, imagine that speaker "A" is talking but a pause > 200ms is observed. When speaker “A” paused for more than 200 milliseconds, we checked whether individual “B” stayed silent; if “B” remained quiet and “A” continued speaking afterward, “A”’s speech segments were combined into a single turn. Moreover, speech units from speakers A and B can occur at the same time (i.e., they overlap). Overlaps could represent an interruption or a backchannel; however, such a distinction was not made here [24]. Both onsets of overlaps and onsets of turns constituted a turn switch. Turn duration was calculated, and revealed that 72% of the turn lasted up to 10 seconds, 22% extended from 10 to 20 seconds, while a minority, comprising only 6%, continued up to 30 seconds. 70% of the turn switches were attributed to overlaps and the remaining 30% to the onsets of turns.

### Wavelet analysis

**Fig 2:**
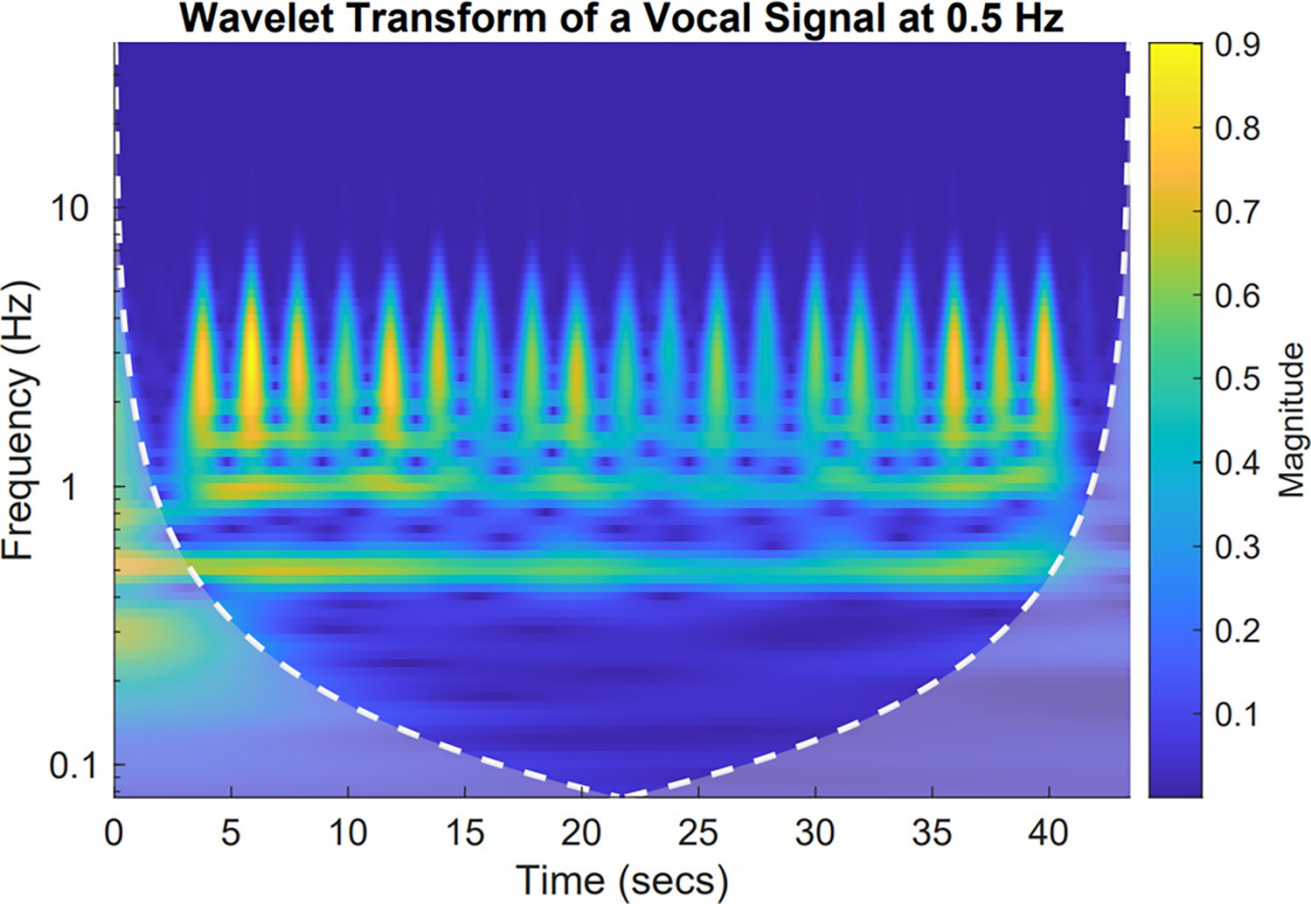
Wavelet transform of a vocal signal at 0.5 Hz. The wavelet transforms represents one person’s voice saying “OK” every 2 seconds (0.5 Hz). The x-axis shows the time in seconds. The y-axis represents the different timescale expressed in frequency (Hz)(i.e., where the 1 Hz = 1/period = 1/1 sec). The main frequency of the voice is represented with the continuous yellow line at 0.5 Hz while the consecutive burst reflects the moment when frequency modification occurs (i.e., when the person said, “OK”).

Studying social interaction involves analyzing different types of signals (i.e., different modalities), such as movement and speech signals. These signals are known to oscillate on multiple timescales, from faster ones (syllable rate) to longer ones (movement duration, turn duration) [22, 41, 67]. Actually, studies suggested that co-speech gestures and speech are organized in a complex, hierarchical manner, where shorter timescale patterns are nested within longer timescales [67]. This means that synchronization could change over the time course of the interaction, making those time series non-stationary [75, 76]. While cross-correlation analyses are commonly used to study interpersonal coordination [77], these methods assume stationarity in the signals [76]. This assumption makes them inadequate for capturing the dynamic phenomena that occur during conversations [2]. To overcome this, wavelet-analysis methods have been recently used in many studies on synchronization [41, 59, 60, 67]. This validated method allows one to investigate complex and non-stationary time series with multiple frequencies occurring at the same time [41, 42]. The analysis resulting from the wavelet transform is then mapped onto a time-frequency plane and illustrated in a wavelet power plot [42]. Fig 2 provides an example of frequency modifications observed in a speech envelope signal.

A Cross-wavelet transform is then employed to evaluate the dynamic interaction implying two different time series. Cross-wavelet wavelet analyses allow giving information about the coherence and relative phase of two signals. The coherence measures the degree of similarity between two-time series at each timescale on a range from 0 to 1. The coherence of 1 reflects perfect synchronization between the two-time series, while 0 characterizes no synchronization. The relative phase captures and quantifies the pattern of synchronization between these two components and can also determine the transition from one behavior to another [42]. Coordination where the two signals are moving together at the same time, has a relative phase of 0° meaning both are in-phase. On the opposite, if people are moving in alternation, their activity time series will be in anti-phase and will have a relative phase angle of 180° [26, 42, 78].

In the current study, cross-wavelet transforms were calculated for each dyad using the MATLAB wavelet toolbox. Morlet was used as the mother wavelet. We extracted the coherence and relative phase values for all the selected sessions across 23 timescale ranges. We extracted the coherence and relative phase values for 23 ranges of timescales ranging from 0.125 s to 30 s (0.125–0.25, 0.25–0.375, 0.375–0.5, 0.5–1, 1–2, 2–3, 3–4, 4–5, 5–6, 6–7, 7–8, 8–9, 9–10, 10–12, 12–14, 14–16, 16–18, 18–20, 20–22, 22–24, 24–26, 26–28, 28–30). These intervals were selected as they represent relevant fine-grained timescales for both speech and movements [59, 67].

The mean of the coherence and the circular mean of the relative phase at these subsidiary timescales were extracted to evaluate patterns and degree of body motor coordination, respectively, and submitted to statistical analyses.

#### Surrogate data

Surrogate data were generated to form control condition estimates to evaluate whether the degree and the pattern of coordination at the different timescales were significantly different from that expected by chance [41].

For interpersonal analyses, between-subject shuffling was applied to create these surrogate data. It consists of permuting each participant’s whole session data, irrespective of session order and participant, creating artificial interaction or “pseudo-dyads”. In this study, we combined Participant 1 from a specific dyad with Participant 2 from all remaining dyads and takes. All possible combinations were extracted, resulting in 942 random pseudo-dyads. When time series were of unequal length, the longer time series was truncated to the length of the shorter series [79].

For intrapersonal analyses, within-subject shuffling (i.e., segment shuffling) was applied to create the surrogate data. This process consists of splitting individual time series into small segments which are then permuted in time but keeping the subject structure intact. In the current study, we chose a segment length equal to 200ms as intrapersonal synchrony can be found at higher frequencies [80, 81].

Overall, the same wavelet coherence and phase analyses were conducted over all possible combinations of pseudo-dyads and then averaged at the dyad level for further statistical analyses.

## Results

The results section is divided into two major parts. The first focuses on intrapersonal analyses, examining both degrees and patterns of synchronization between the speech and the gesture (unimodal and multimodal). The second focuses on interpersonal analyses, examining both degrees and patterns of synchronization between the speech and the gesture (unimodal and multimodal). The term “unimodal” focuses on “movement vs. movement” analyses (e.g., head vs. head, head vs. wrist) or “voice vs. voice” analyses. In contrast, the term “multimodal” involves the joint analysis of movement and voice modalities (e.g., head vs. voice; wrist vs. voice). To analyze degrees and patterns of synchronization, separate analyses of variance (ANOVA) were conducted on the mean coherence and the circular mean relative phase respectively. Condition (Experimental, Virtual) and Timescales (ranging from 0.125 s to 30 s) were chosen as variables. Moreover, depending on whether the analyses were unimodal or multimodal, Modalities (such as Head vs. Head, Head vs. Wrist, and Head vs. Voice, etc.) were included as a third variable. For all statistical analyses, Greenhouse-Geisser adjustments for violations of sphericity were made as necessary. For post hoc analyses and pairwise comparisons, Bonferroni correction was implemented to determine significant differences between individual means.

### What degrees and patterns of synchrony are observed within participants (intrapersonal analyses)?

#### Unimodal movement vs. Movement analyses

The unimodal analyses focused exclusively on one modality of “Wrist vs. Head”. Therefore, a two-way repeated ANOVA was conducted on both mean coherence and mean relative phase with Condition and Timescales as within-variables.

#### Degree of coherence

**Fig 3:**
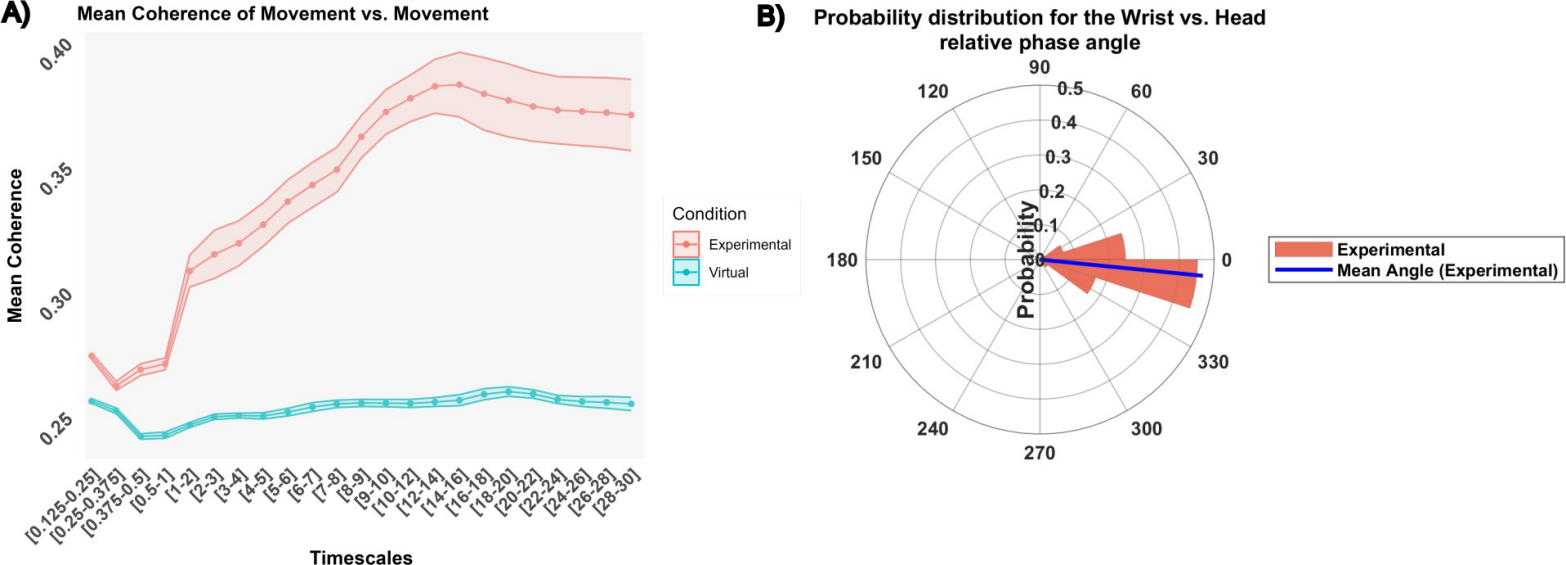
Intrapersonal degree and pattern of synchronization: Unimodal analyses. (A) Differences in mean coherence between the experimental condition (red) and the virtual condition (blue), across different timescales. Mean and standard deviation are represented with dots and colored ribbons respectively. Grey areas depict significant differences between conditions. Here, all timescales are significant. (B) A circular histogram depicting the probability distribution for the experimental condition and ranges timescale where wavelet significantly differed from chance. The blue line represents the mean phase angle. Unimodal wrist vs. head relative phase angle oscillates around -6°, the head leading the wrist on average.

The ANOVA revealed a significant main effect of Condition (*F*(1, 13) = 154.95, *p* < .0001, *η^2^* = 0.73) on the mean coherence. Post-hoc analysis highlighted a significantly higher mean coherence for the experimental condition (*M* = 0.34) compared to the virtual condition (*M* = 0.25) (Fig 3A). Therefore, the subsequent analysis focused on the experimental group and found a main effect of Timescales (*F*(22,286) = 25.89, *p* < .0001, *η^2^* = 0.54). The highest mean coherence was found in the 12–16 s range (*M* = 0.38) while the lowest was found in the 0.25–0.5 range (*M* = 0.26). No effects of Modality were found to be significant.

#### Pattern of synchronization

The ANOVA was performed within all timescales. It revealed a significant interaction effect between Timescales and Condition (*F*(22, 286) = 1.99, *p* < .05, *η^2^* = 0.062). Post-hoc analysis highlighted an effect of Condition in the time intervals 0.375–0.5, 0.5–1, 1–2, and 4–5 s. Further descriptive analysis focused on these specific timescales and involved wrapping the data around 360° to acknowledge the inherent cyclic nature of the data and enhance visualization. Circular plots were handled using the toolbox for circular statistics with Matlab [82]. It demonstrated that the angle where oscillations were most concentrated (the peak of the probability distribution) was identified to be 342° (i.e. -18° in the range [-180°-180°]) and that the average angle direction was found at -6°. On average, intrapersonally, the head tended to precede the wrist, indicating an in-phase lead (Fig 3B).

### Multimodal voice vs. Movement analyses

The multimodal analyses between voice and movement focused on two modalities “Wrist vs. Voice”, and “Head vs. Voice”. Therefore, a three-way repeated ANOVA was conducted on both mean coherence and mean relative phase, with Condition and Timescales, and Modalities (added here as a third variable).

#### Degree of coherence

**Fig 4:**
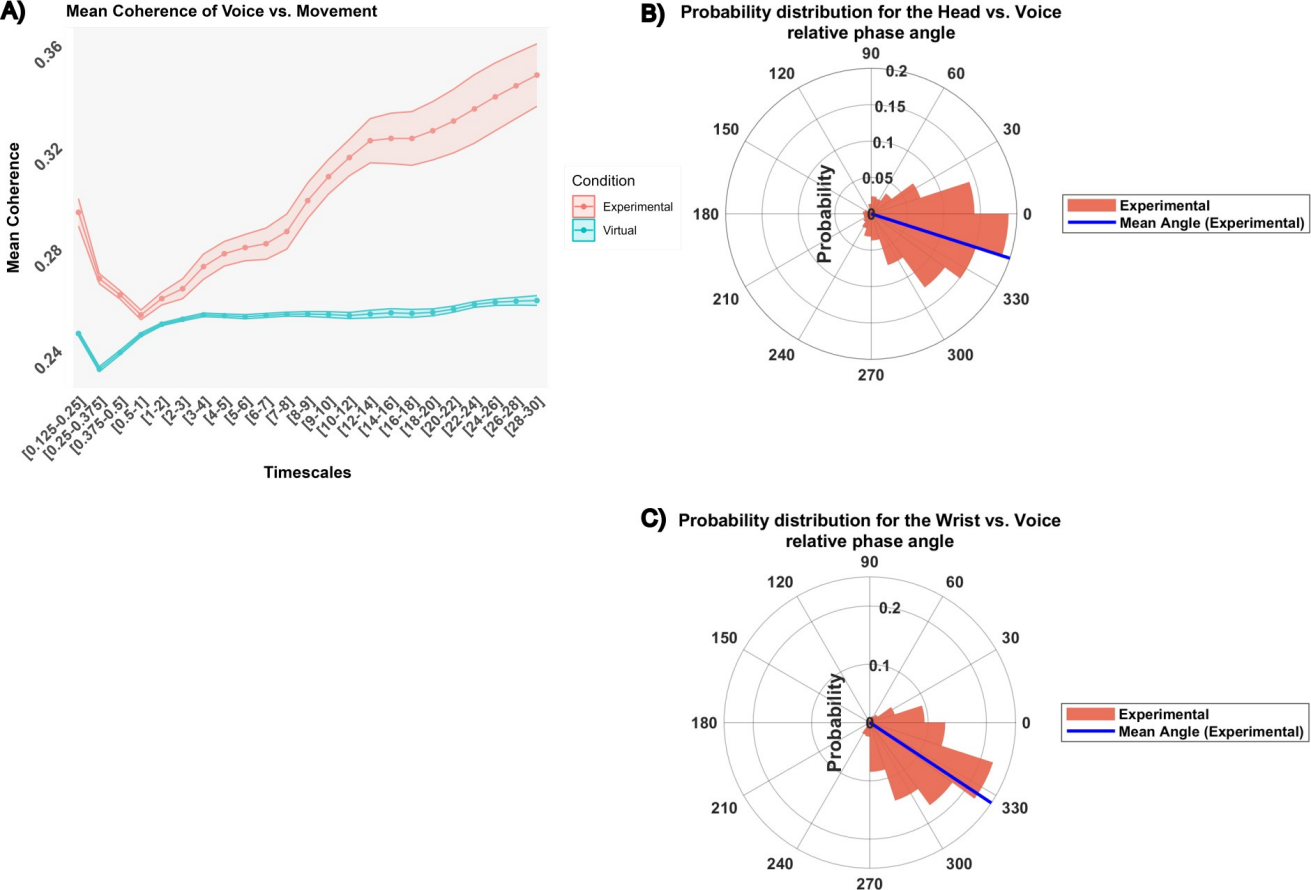
Intrapersonal degree and pattern of synchronization: Multimodal analyses. (A) differences in mean coherence between the experimental condition (red) and the virtual condition (blue), across different timescales. Mean and standard deviation are represented with dots and colored ribbons respectively. Grey areas depict significant differences between conditions. Here, all timescales are significant. (B) and (C) multimodal Head vs. Voice and Wrist vs. Voice relative phase angle respectively. The circular histograms depict the probability distribution for the experimental condition for timescales where wavelet significantly differed from chance. The blue line represents the mean phase angle. Head vs. voice relative phase angle oscillates around -18°, the voice leading the head on average. The wrist vs. voice relative phase angle oscillates around -34°, the voice leading the wrist on average.

The ANOVA revealed a significant interaction effect between Condition and Timescales (*F*(22, 286) = 16.66, *p* < .0001, *η^2^* = 0.15). In spite of the interaction, further analysis highlighted a statistically significant effect of condition at all levels of Timescales, with a higher mean coherence for the experimental condition (*M* = 0.29) compared to the virtual condition (*M* = 0.25) (Fig 4A). Therefore, the subsequent analysis focused on the experimental condition and found a main effect of Timescales (*F*(22,286) = 22.54, *p* < .0001, *η^2^* = 0.32). The highest mean coherence was found in the 26–28 s range (M = 0.34) while the lowest was found in the 0.5–2 s range (*M* = 0.25). No effects of Modality were found to be significant.

#### Pattern of synchronization

The ANOVA was performed within all timescales. It revealed a significant interaction effect between Condition and Timescales (*F*(22, 286) = 3.79, *p* < .0001, *η^2^* = 0.06). The interaction effect underscored a difference in the mean circular relative phase between the Experimental and Virtual conditions, especially within the 3–18 range (*M*_*experimental*_ = -23.54, *M*_*virtual*_ = 0.229), the 20–26 range (*M*_*experimental*_ = -15.34, *M*_*virtual*_ = 2.54), and the 28–30 range (*M*_*experimental*_ = -16.6, *M*_*virtual*_ = -0.79). Further descriptive analysis indicated that the angle where oscillations were most concentrated (the peak of the probability distribution) was identified to be 342° (i.e. -18° on the range [-180°-180°]) for the Head vs. Voice angles, and 324° (i.e. -36° on the range [-180°-180°]) for the Wrist vs. Voice angles. The average angle direction was found at -18° for the Head vs. Voice angle, highlighting that intrapersonally, the voice tended to precede the head, indicating an in-phase lead (Fig 4B). The average angle direction was found at -34° for the Wrist vs. Voice angle, highlighting that intrapersonally, the voice tended to lead the wrist, indicating an in-phase lead (Fig 4C).

### What degrees and patterns of synchrony are observed between participants (interpersonal analyses)?

#### Unimodal voice vs. Voice analyses

The unimodal analyses focused exclusively on one modality of “Voice vs. Voice”. Therefore, a two-way ANOVA was conducted on both mean coherence and mean relative phase, with Condition as the between-subject variable and Timescales as the within-subject variable.

#### Degree of coherence

**Fig 5:**
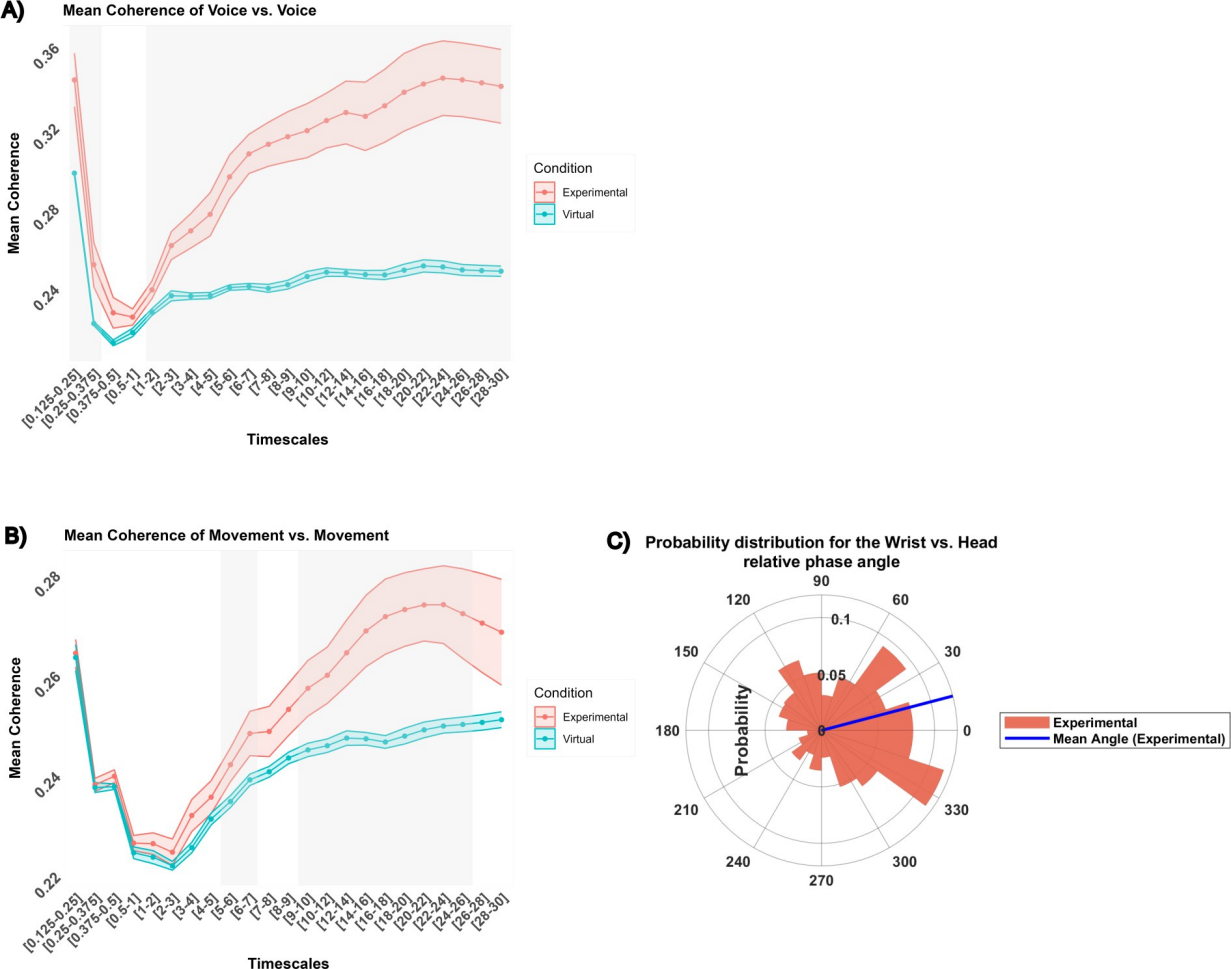
Interpersonal degree and pattern of synchronization: Unimodal analyses. (A) differences in mean coherence between the experimental condition (red) and the virtual condition (blue), across different timescales. Mean and standard deviation are represented with dots and colored ribbons respectively. Grey areas depict significant differences between conditions. Here, the significative timescales are 0.125–0.375, and 1–30 for (A); 5–7 and 9–26 for (B). (C) A circular histogram depicting the probability distribution for the experimental condition and ranges timescale where wavelet significantly differed from chance. The blue line represents the mean phase angle. Unimodal wrist vs. head relative phase angle oscillates around 14°, the wrist leading the head on average.

The ANOVA revealed a significant interaction between Condition and Timescales (*F*(22, 572) = 6.75, *p* < .0001, *η^2^* = 0.14). The interaction effect indicated that the Experimental group exhibited a higher mean coherence in comparison to the virtual dyads within all ranges except 0.375–1 (Fig 5A). The subsequent analysis focused on the Experimental condition on ranges where wavelet coherence was significantly higher than chance. An ANOVA revealed a significant effect of Timescales (*F*(20,260) = 9.01, *p* < .0001, *η^2^* = 0.28). The highest mean coherence was found in the 22–26, and 0.125–0.25 s ranges (*M* = 0.34) while the lowest was found in the 0.25–0.375, and 1–3 s ranges (*M* = 0.25). No effects of Modality were found to be significant.

#### Pattern of synchronization

One may note that since the voice vs. voice comparison is between the same modalities (voice), further analysis wouldn’t clarify which modality is leading. Such an analysis would only address which participant’s voice was leading the other. Since this is likely random within and across dyads, no pattern of synchronization analyses was conducted unimodal voice vs. voice coordination.

#### Unimodal movement vs. Movement analyses

The unimodal movement analyses focused on three modalities, including “Head vs. Head”, “Wrist vs. Wrist”, and “Wrist vs. Head”. Therefore, a three-way ANOVA was conducted on both mean coherence and mean relative phase, with Condition as the between-subject variable, Timescales and Modalities as within-subject variables.

#### Degree of coherence

The ANOVA revealed a significant interaction effect between Condition and Timescales (*F*(22, 572) = 2.76, *p* < .0001, *η^2^* = 0.03). The interaction effect indicated that the Experimental group exhibited a higher mean coherence in comparison to the virtual dyads within specific ranges: 5–7 s (*M*_*experimental*_ = 0.24, *M*_*virtual*_ = 0.24), and 9–26 s (*M*_*experimental*_ = 0.26, *M*_*virtual*_ = 0.23). Within these specific timescales, the coherence of the experimental dyads was significantly above the one obtained from chance (virtual condition), irrespective of the modality (Fig 5B). A subsequent analysis focused on the Experimental condition on ranges where wavelet coherence was significantly higher than chance. An ANOVA revealed a significant effect of Timescales (*F*(9, 117) = 4.85, *p* < .0001, *η^2^* = 0.06). The highest mean coherence was found in the 18–22 range (*M* = 0.27) while the lowest was found in the 5–7 s range (*M* = 0.24). No effects of Modality were found to be significant.

#### Pattern of synchronization

The three-way ANOVA was performed on ranges where wavelet coherence was significantly different from chance.

The ANOVA revealed significant main effects for Condition (*F*(1, 26) = 5.16, *p* = .032, *η^2^* = 0.08). The angle where oscillations were most concentrated (the peak of the probability distribution) was identified to be 324° (i.e. -36° in the range [-180°-180°]), and the average angle direction was found at 14°. On average, interpersonally, the wrist tended to lead the head, indicating an in-phase lead (Fig 5C).

#### Multimodal voice vs. Movement analyses

The multimodal voice/movement analyses focused on two modalities, including “Head vs. Voice”, and “Wrist vs. Voice”. Therefore, a three-way ANOVA was conducted on both mean coherence and mean relative phase, with Condition as the between-subject variable and Timescales and Modalities as within-subject variables.

#### Degree of coherence

**Fig 6:**
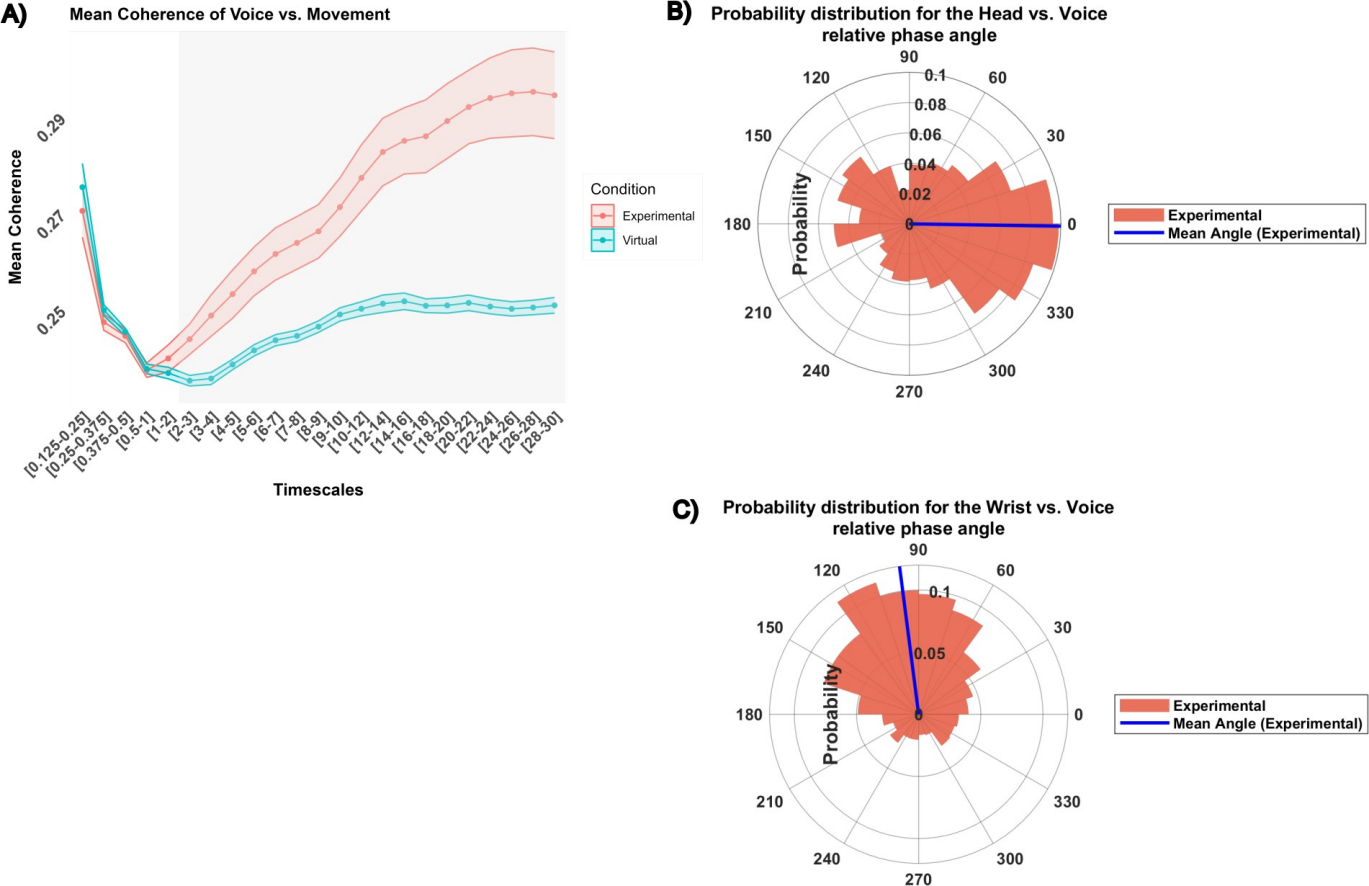
Interpersonal degree and pattern of synchronization: Multimodal analyses. (A) differences in mean coherence between the experimental condition (red) and the virtual condition (blue), across different timescales. Mean and standard deviation are represented with dots and colored ribbons respectively. Grey areas depict significant differences between conditions. Here, the significative timescales are 2-30s. (B) and (C) multimodal head vs. voice and wrist vs. voice relative phase angle respectively. The circular histograms depict the probability distribution for the experimental condition for timescales where wavelet significantly differed from chance. The blue line represents the mean phase angle. Head vs. voice relative phase angle oscillates around 5°, the head leading the voice on average. The wrist vs. voice relative phase angle oscillates around 100°, the voice leading the wrist on average.

The ANOVA revealed a significant interaction between Condition and Timescales (*F*(22, 572) = 9.45, *p* < .0001, *η^2^* = 0.12). Irrespective of the modalities, the interaction effect indicated that the Experimental group exhibited a higher mean coherence in comparison to the virtual dyads on timescales ranging from 2 to 30 s (Fig 6A).

The subsequent analysis focused on the Experimental condition on ranges where wavelet coherence was significantly higher than chance. An ANOVA revealed a significant effect of Timescales (*F*(14, 182) = 11.01, *p* < .0001, *η^2^* = 0.20) and Modalities (*F*(1,13) = 4.85, *p* < .05, *η^2^* = 0.052). The highest mean coherence was found in the 26–30 s range (*M* = 0.29) while the lowest was found in the 2–5 s range (*M* = 0.25). In addition, the coherence between Head vs. Voice (*M* = 0.28) was significantly higher than the coherence between Wrist vs. Voice (*M* = 0.27).

#### Pattern of synchronization

The three-way ANOVA was performed on ranges where wavelet coherence was significantly different from chance.

The ANOVA revealed a significant main effect for Condition (*F*(1, 26) = 13.47, *p* = 0.001, *η^2^* = 0.13). Further descriptive analysis indicated that the angle where oscillations were most concentrated (the peak of the probability distribution) was identified to be 342° (i.e., -18° on the range [-180°-180°]) for the Head vs. Voice angles, and 108° for the Wrist vs. Voice angles. The average angle direction was found at -0.9° for the Head vs. Voice, highlighting that interpersonally, the voice tended to lead the head, indicating an in-phase lead (Fig 6B). The average angle direction was found at 97° for the Wrist vs. Voice angle, highlighting that interpersonally, the voice tended to lead the wrist, indicating an anti-phase lead (Fig 6C).

## Discussion

The current study was designed to enrich our full picture of the multimodal behavior dynamics observed during social interaction, at the intrapersonal and interpersonal levels. We concentrated our attention on specific modalities including the voice and head/wrist non-verbal gestures performed during an unstructured conversational task. We used the cross-wavelet coherence and relative phase to investigate the degree and pattern of synchronization of these modalities across different timescales.

For our first focus, intrapersonal synchronization, we observed, as expected, higher-than-chance self-coherence both between the head and wrist movements and between the participant voice and movements but importantly also found that self-synchronization occurred at all the interaction timescales. In exploring the pattern of these synchronizations, we found that intrapersonal synchronization exhibited an in-phase correlation in both unimodal movement synchronization and multimodal gesture-speech synchronization.

For our second focus, interpersonal synchronization, we found higher-than-chance unimodal movement-movement and voice-voice coordination, as we hypothesized but also extended previous findings by highlighting higher-than-chance multimodal synchronization between the voice and the movement of interlocutors at specific conversation timescales. The analyses of the relative phase highlighted an in-phase relationship with unimodal movement synchronization as well as for multimodal head vs. voice, but an anti-phase pattern was found between the wrist and the voice interpersonally.

### Intrapersonal degree of synchrony

A primary focus of this study was to analyze the degree and pattern of synchronization observed at the intrapersonal level, both in terms of unimodal and multimodal synchronization. Our results demonstrated higher-than-chance unimodal synchronization between the participant’s wrist and head movements and found similar synchronization in the multimodal coordination between the participant’s head/wrist movements and voice. These findings are consistent with what might be expected from one gesticulated during speech and are supported by past research. For example, Hadar et al. [83] recorded participants’ head movements during conversations and discovered that the head maintains nearly constant movement during speech. It is not surprising that head movements match closely with speech, as speakers commonly use head movements to emphasize key points or intensify words [14]. Our results also relate to Tuite’s [84] Rhythmical Pulse Hypothesis which postulates that the gestural stroke of either manual or non-manual gesture coincides with the intonation peak of spoken language [10]. This theory was illustrated in the literature on gesture-speech synchronization in which it is highly demonstrated that manual gestures co-occur in time with the suprasegmental properties of speech such as intonation and rhythm [10]. This process was depicted in the study of Pouw et al. [63] which demonstrated how rhythmic arm movements influenced vocalization acoustics by amplifying the amplitude envelope of speech.

Moreover, our results revealed that synchronization, whether unimodal or multimodal, occurs throughout all the conversational timescales, from faster time intervals at 0.125 seconds to longer ones at 30 seconds. Underlying the observation is the view that naturalistic settings such as conversations are made up of the succession of vocal exchanges between individuals, referred to as turns [85].

Considering that speakers take the floor one at a time, one speaker’s turn will consist of utterances, which are sequences of words, further broken down into sequences of syllables. Hence, at the intrapersonal level, these linguistics hierarchical events involve multiple embedded timescales [67, 86]. For instance, in an experiment where a participant had to retell a cartoon, Pouw and Dixon [67] found that speech and hand gestures converged at the periodicity relevant to syllable completion times (0.125 sec to 0.5 sec), gesture completion times (0.5 sec to 2 sec), and sentence completion times (2–6 sec). These shared temporal structures between speech and gesture seem consistent with a subset of our results, notably for the coherence found at the fastest timescales (0.125–0.5 sec), likely illustrating the syllable duration. Indeed, this latter vocal feature is known for its consistency and stability across language, with particular oscillation around 200ms [87].

However, our results revealed the highest coherence for the longest timescales (26–28 sec), a finding not accounted for in previous speech/gesture synchronization studies. One potential explanation lies in the fact that participants’ turns are highly dependent on the interpersonal dynamics of the interaction, which is also influenced by the type of conversational task employed. Introducing a simple modeling framework simulating the dynamics of speakers’ behaviors across different task contexts, Miao et al. [88] highlighted that small changes in the task configuration (i.e., a topic or goal change) may indeed modify the structure of turns. These views would effectively match the interpersonal synergy theory, which suggests that a conversation, and therefore the turn it constitutes, cannot be fully understood at this individual component level but must be integrated within the whole conversation organization that is shaped by the task constraints. Supporting this theory, a study by Dideriksen et al. [89] showed how task demands (whether demanding a high or a low level of precision) leverage the rate and level of conversational entrainment, to foster mutual comprehension.

In the current study, the participants were engaged in long unstructured conversations that don’t necessarily involve rapid exchanges like question-answer sessions. We noted that around 70% of the turns lasted up to 10 seconds, with 20% extending to 20 seconds. We believe this relatively slow rhythm of turn could encompass discussions that delve into deeper and more open-ended subjects, such as the sharing of personal experiences, or more nuanced exploration of ideas. Moreover, Yuan et al. [90] found that the topic and the relationship between speakers could affect turn length and speaking rate, with longer turns observed between strangers. The authors attributed these longer turns to the potential formality of interactions and the absence of shared knowledge between strangers. This explanation could account for our results, as participants in our study were unfamiliar with each other.

### Intrapersonal pattern of synchrony

Our unimodal relative phase results indicate that the head and the wrist tend to be in-phase, with the head leading the wrist on specific timescales of 0.375–2 s and 4–5 s whereas the multimodal coordination of voice and body, reveal higher than chance in-phase relative phase on the time intervals between 3–18, 20–26 and 28–30 s. This in-phase synchronization underscores the intimate coupling between an individual’s gesture and speech. Moreover, on average the voice leads the wrist and head movements, as indicated by the mean relative phase of -34° and -18°, respectively.

On the face of it, these results seem at odds with a previous study by Pouw, Harrison et al. [69] that highlighted that repetitive arm beat movements tend to entrain phonation. The authors explained this finding by the physical coupling between arm movement and speech. When making a gesture, a force will be produced which will increase alveolar lung pressure. This increase will modulate the laryngeal pressure, leading to changes in the amplitude and intensity of vocalization. This process holds for emphasized gestures, such as simple and fast arm movements (i.e., beat gestures), as they demand greater force production [11, 69]. However, the differences in the types of gestures being analyzed could account for the disparities with our results. Indeed, we analyzed the entire movement time series without systematic annotation of a specific movement. It resulted in a wider range of gestures, some of them possibly less physically effortless, such as simple wrist movements or natural oscillations. Results on temporal alignment are therefore less precise, and a possible phase shift could have happened between any kind of postural and voice oscillation. This explanation applies to all our results on the relative phase.

### Interpersonal degree of synchrony

The second focus of this study aimed to analyze the degree and pattern of synchronization observed at the interpersonal level, in terms of both unimodal and multimodal synchronization—between the voices and the bodies of the two speakers as well as the voice of one speaker and body of the other. Unimodal voice analyses revealed higher-than-chance coherence between participants’ voices on all temporal ranges except for 0.375-1sec. These observations are consistent with Wilson and Wilson’s [22] dynamic model of turn-taking which proposed that speakers’ oscillatory cycles are established by their syllable rate which rhythmically entrain the listeners’ oscillators. Moreover, Manson and collaborators [91] examined dyadic synchronization in vocal characteristics and found that mean syllable duration (i.e., speech rate) converged through the interaction. When the two speakers talk one at a time, syllable duration convergence might not be indicative of synchronization. However, daily conversations are not that simple and often involve overlaps, whether when speakers change turns or use backchannels and interruptions [92]. In our study, 70% of the turn switches were found to be overlaps. Therefore, it is suitable to believe that synchronization can occur at the syllable duration for the overlapping part of the conversation. In addition, unimodal voice synchronization is likewise determined in larger temporal intervals, with a tendency among speakers to coordinate their voice at the turn level (i.e., speakers are more similar to each other at turn exchanges) [47]. These observations potentially explain our findings of high coherence observed for shorter timescales which are associated with syllable durations, as well as high coherence for longer timescales which are representative of turn durations.

Regarding unimodal movement-movement analyses, results revealed higher-than-chance coherence between participants’ movements, with specific temporal ranges showing greater synchronization. More precisely, the participants synchronized their movements at a middle timescale of 5–7 s and at a slower timescale between 9 s and 26 s. While consistent with past literature on existing bodily synchronization, our findings diverge on the associated periodicity. In their Knock-knock jokes task, Schmidt et al. [41] found a higher-than-expected level of synchronization between the global quantity of movement of the two interactants. They identified the moment where high synchronization happened to be every 1.5 s, when the speaking turn occurs, as well as every 6 s, at the end of the joke. However, knock-knock jokes are a highly structured conversation, and a degree of synchronization could have emerged from this inherent rhythmic organization. In a recent paper, Schmidt et al. [93] overcame this by analyzing synchronization within an interview, a less structured task. They observed synchronization occurring over longer timescales ranging from 10 to 16 s, consistent with the timing of the interview questions. Moreover, they found synchronization even in the absence of visual cues. They discussed this outcome, stating that interpersonal coordination of speech rhythms provides the basis for interpersonal synchronization. For this reason, we posit that distinct turn rhythms significantly impact interpersonal synchronization, similar to our explanation for intrapersonal coordination. This point of view is supported by a study conducted by Fujiwara et al. [59], wherein dyads participated in unstructured conversation. They found the highest coherence to increase for the longer timescales 2–40 s compared to faster ones 0.25–2 s. Although the author did not extract any information about the turn-taking space, they suggest these slow rhythms to be representative of our everyday conversation. These findings corroborate our results, with the highest synchronization observed over longer timescales. Moreover, the task used in our study also resembles daily conversation which might explain this similarity.

Concerning multimodal coherence of the voice of one speaker and the body of the other, we interestingly found similar results to those of unimodal coherence. The voice of one participant and the movements of the other participant are synchronized above the chance level for the middle and slower timescales between 2 s and 30 s. These results are in line with our explanation for the unimodal coherence, supporting that the vocal features involved in the sentences and turn duration indeed seem of major importance in multimodal coherence. Another possible explanation of these specific timescales may be attributed to backchanneling, a fundamental feature of multimodal voice/gesture synchronization. Backchannels are described as feedback produced simultaneously with speech to provide speakers with real-time information about how their turn is being received [94]. According to previous studies, vocal and nonverbal backchannels occur at the same time, with vocal cues occurring every 9 s and nonverbal cues every 6 s [89, 95, 96]. Based on our results, it is possible that high synchronization found between the voice and movements, around 6 and 9 s, depicts the use of backchannels.

### Interpersonal pattern of synchrony

Our results on the unimodal pattern of synchrony indicate a relative phase higher than chance on the time intervals between 3–18, 20–26 and 28–30 s. While the wrist leads in-phase on average, there are notable instances where the head leads (as indicated by the probability peak at -36°).

For the multimodal analyses of the voice of one speaker and body of the other, a relative phase higher than chance was found on the time intervals between 2 s and 30 s. On average, the voice leads wrist movement in antiphase and the voice leads the head in-phase. We believe the anti-phase relationship found between the wrist and the voice of the participant to be representative of the turn-taking nature of the task. Indeed, while taking the floor, the speaker will actively use their hands to emphasize their speech. Conversely, the listener tends to remain comparatively still [97]. In the same way, the in-phase relationship between the voice and the head could also reflect the dynamics of the conversation, notably highlighting the feedback nature of the listener’s head movements and vocalizations. This explanation is coherent with previous studies which observed that the feedback nods produced by the listeners were close in time to their corresponding speech, preceding it by ~175-400ms [98, 99]. The authors proposed that vocal responses and nonverbal head movements serve an interpersonal function, where the listener addresses feedback to the speaker. In other words, while a participant is speaking, the listener will likely produce backchannels in the form of head nods or soft vocalization to provide information about its involvement, without disturbing the interlocutor’s speech. Low-amplitude single nods were indeed found to happen in phase with speakers’ stressed syllables [100]. Moreover, this would also account for the in-phase relationship between the wrist and the head of the participants that we observed, as wrist movement typically occurs during the speaking turn.

Table 1 summarizes the major findings of this study in relation to previous results. Overall, for the intrapersonal and interpersonal analyses, our findings support the idea of self-coordination between the speaker’s voice and its movements at all timescales of the conversation, including the syllable rate. At the interpersonal level, synchronization was found at specific timescales, which we believe are relevant to the turn-taking dynamics of the conversation. Notably, coordination seems to match the speaker’s turn duration. Moreover, because we found unimodal and multimodal coordination to append approximatively on the same timescale, we assume these synchronizations to rely mostly on the auditory channel, specifically on speech rhythms [93]. This would verify previous studies that emphasized that verbal information only is sufficient for creating spontaneously coordinated movements between two people talking to each other [41, 101]. In addition, we highlighted in-phase relationships for intrapersonal synchronization as well as some anti-phase relationships for interpersonal synchronization, which certainly accounted for the turn-taking dynamics of the interaction. Moreover, our results suggest that some intrapersonal patterns of relative phase synchronization coincide with interpersonal ones. Actually, intrapersonal coupling between the head and the voice hit the same peak of oscillation as interpersonal coupling between the head and the wrist (i.e. -36°). In the same view, intrapersonal coupling between the voice and the wrist, and between the head and the wrist also shares the same peak of oscillation as interpersonal coupling between the head and the voice (i.e. -18°). This similarity seems to indicate that the same mechanism underlies both intrapersonal and interpersonal communication and, hence, provides support for the behavioral dynamics’ perspective that the same dynamical processes of self-organization that constrain physical oscillators govern coordination at the behavioral and social scales of nature [93].

**Table 1:** Overview of key findings in previous research on intrapersonal and interpersonal synchronization relative to our results.

		Past Research	Results of the current study
**Intrapersonal synchronization**	**Degree of synchro**	Gestures co-occur in time with the supra-segmental properties of speech. →Rhythmical Pulse Hypothesis as explained in [10].	Multimodal Coherence > chance for all conversational timescales (0.125-30s).
		One individual turn consists of utterances, which are sequences of words, further broken down into sequences of syllables = embedded timescales. →Convergence at the syllable rate (~200ms) [67, 85–88]	Unimodal and multimodal synchronization was observed around 0.2s, highlighting the fast oscillation likely occurring at the syllable rate.
		Participant’s turns are highly dependent on the interpersonal dynamics of the interaction, which is also influenced by the type of conversational task employed. →Interpersonal synergy theory [88, 32, 89, 90].	This could explain the longer timescales found for the multimodal coherence.
	**Pattern of synchro**	Arm tend to entrain phonation →Gesture-speech physics theory [69].	This view does not support our result of the voice leading the wrist and head inphase for multimodal synchronization.
**Interpersonal synchronization**	**Degree of synchro**	Linguistic features, such as syllable rate entrain voices →Wilson and Wilson’s (2005) theory [22; 91].	Voice vs. Voice unimodal synchronization was observed around 200ms, highlighting the fast oscillation likely occurring at the syllable rate.
		Movements are synchronized at longer timescales and are dependent upon speech rhythms, such as turn duration, which is context-dependent [59, 93].	This could explain the longer timescales found for the multimodal and unimodal coherence.
		Vocal and nonverbal backchannels occur at the same time, with vocal cues occurring every 9 s and nonverbal cues every 6 s [89, 96].	Support our results for multimodal voice vs. movement synchronization found around medium timescales (~5-10s).
	**Pattern of synchro**	While taking the floor, the speaker will actively use their hands to emphasize their speech. Conversely, the listener tends to remain comparatively still [97].	We believe these dynamics illustrate the turn-taking process and could account for the antiphase relationship between the voice and the wrist.
		Vocal responses and nonverbal head movements serve an interpersonal function, where the listener addresses feedback to the speaker [98, 99]. →Low-amplitude single nods were found to happen in phase with speakers stressed syllables [100].	This would account for the in-phase relationship between the head and the voice. As wrist movement typically occurs during the speaking turn, it would also explain the inphase relationship between the wrist and the voice.

## Limitations and directions for future research

While our results provide insight into the multimodal dynamics of social interaction, there are also limitations to consider. First, the dataset allowed us to use only a small number of dyads, which could have lowered the statistical power of our results and complicated the generalization of our findings. Second, while all the dyads were different in their composition, one participant engaged in all the interactions (i.e., participated in all conversations). This could have influenced our findings on synchronization as this consistent participant could have led to a uniform pattern of synchronization across timescales and all interactions. Third, the generation of intrapersonal surrogate time series involved shuffling within subjects, using a specific segment length of 200ms that may not have captured all synchronization patterns comprehensively. While the choice of this segment size was made based on the prior knowledge of the tendency of speakers to synchronize at the syllable duration (around 200ms), it could have overlooked longer synchronization patterns [80]. We suggest future research to explore surrogate methods capable of representing both short and long-term synchrony more effectively. Fourth, while we extracted turn duration from our manual annotation combined with a MATLAB script, other automatic methods could have been employed to obtain more detailed information about participant sentences and syllable length. Fifth, we believe that further vocal and gestural annotation could be beneficial in identifying and understanding the causal relationship between specific vocal features (i.e., such as turn, and backchannels) and corresponding gestures. This deeper classification could indeed provide more comprehensive insights into how multimodality between the voice and movements interact and influence each other during social interaction. Then, while cross-wavelet coherence measures time coherence, it may not fully capture the dynamics (whether speech or movements) that are not closely aligned in time. In support of this assumption is the interpersonal synergy view, which highlights that behaviors may not always synchronize but complement each other (e.g., one speaks while the other listens) in a manner that ensures the coherence of the global dyadic-system [32]. Finally, as described by Mogan et al. [45], the ability of individuals to synchronize their vocalization and movements helps increase perceived social connection, positive affect, and prosocial behaviors. Extending our findings on multimodal synchronization to a more thorough classification could shed light on which behavior leads to different prosocial behaviors, especially among individuals encountering deficits in social connection, such as in individuals diagnosed with schizophrenia.

## Conclusion

In conclusion, the current study provided evidence of unimodal and multimodal synchronization in unstructured conversation, both at the intrapersonal and interpersonal levels. While intrapersonal coordination relates to specific vocal properties such as syllable duration or backchannel time, interpersonal coordination also seems mediated by some vocal features. Notably, we found the turn-taking dynamics of the interaction to be of particular importance in the observed synchrony, likely to enable the conversation to proceed efficiently. These findings highlight the major contribution of vocal rhythm features, on the specific time interval in which synchronization occurs. Overall, this study extends previous research on interpersonal gesture synchronization and strengthens our knowledge regarding specific speech-gesture synchrony.
